# Editorial: Nutritional modulation of inflammation and insulin resistance

**DOI:** 10.3389/fnut.2023.1181809

**Published:** 2023-03-23

**Authors:** Sandro Massao Hirabara, Renata Gorjao, Rui Curi, Carol Gois Leandro, Gabriel Nasri Marzuca-Nassr

**Affiliations:** ^1^Interdisciplinary Post-graduate Program in Health Sciences, Cruzeiro do Sul University, São Paulo, Brazil; ^2^Immunobiological Production Section, Bioindustrial Center, Butantan Institute, São Paulo, Brazil; ^3^Department of Nutrition, Centro Acadêmico de Vitória, Federal University of Pernambuco, Recife, Brazil; ^4^Universidad de La Frontera, Faculdad de Medicina, Departamento de Ciencias de La Rehabilitación, Temuco, Chile

**Keywords:** nutrients, immunometabolism, insulin signaling, oxidative stress, inflammatory and metabolic diseases, potential biomarker candidates

Several chronic diseases have been associated with increased chronic inflammatory state and insulin resistance conditions, including type 2 diabetes mellitus, metabolic syndrome, cardiovascular diseases, and some types of cancer. Nutrients and various dietary bioactive compounds present an essential role in the modulation of inflammation and insulin sensitivity. However, the molecular mechanisms are not completely known yet.

Several groups have previously demonstrated the importance of nutritional compounds in the modulation of insulin resistance and inflammation (1–5). Thus, the present Research Topic aimed to publish the most recent and relevant studies focused on understanding the effects of nutrients and dietary bioactive compounds on inflammation and/or insulin resistance. Particularly, studies addressing cellular and molecular mechanisms involved in these two disturbances were of interest. The highlight topics for this Research Topic included: (a) the role of nutrients on the development of inflammation and metabolic diseases; (b) the modulation of nutrients and active dietary compounds on inflammation and insulin resistance; (c) the nutritional effects on inflammatory state and insulin signaling pathways; (d) the identification of potential molecular and cellular targets aiming to prevent and/or treat inflammation and related metabolic diseases; and (e) the role of the nutrition on the relationship between inflammatory state and insulin resistance.

The study by Inui et al. investigated the effects of degalactosylated whey protein on lipopolysaccharide (LPS)-induced inflammatory responses in mice in comparison with intact whey protein. Administration of LPS significantly increases plasma tumor necrosis factor-a (TNF- a) and interleukin-1b (IL-1 b) levels, which were significantly suppressed by the administration of degalactosylated whey protein, but not by intact whey protein. The marked increase in the expression of TNF-a and IL-1 b in response to LPS in RAW264.7 cells was significantly suppressed by the application of degalactosylated whey protein. The findings revealed that degalactosylated whey protein has an anti-inflammatory effect and expand the knowledge about the role of degalactosylated whey protein in suppressing inappropriate overactivation of the immune response.

The consumption of a low-protein diet during gestation and lactation has been related to the etiologies of type 2 diabetes, by a mechanism that includes insulin intracellular signalization. Vasconcelos et al. verified that the reduced activation of the Akt_ser473_ found in adult rats caused by a maternal low-protein diet was modulated by mTOR signaling pathway. Impairment in the protein metabolism leads to muscle mass loss, which, in turn, increases the risk of developing related diseases, including type 2 diabetes in adulthood. This study also demonstrated that the long-term changes induced by maternal undernutrition may be regulated by epigenetic mechanisms, such as DNA methylation, histone modification, and non-coding RNAs.

In the work of Ren et al., the authors investigated the potential effects of *Bacillus toyonensis* (Strain SAU-20) on insulin resistance in type 2 diabetes mellitus, since the microbiota has been suggested to modulate insulin resistance and related metabolic diseases, including hepatic steatosis. Obese and type 2 diabetic mice submitted to oral treatment with SAU-20 presented several beneficial effects, including reduced glucose intolerance, insulin resistance, body weight, and fat mass, as well as, decreased hepatic steatosis and ameliorated liver function. These effects were associated with improved lipid profile, reduced oxidative stress markers, and hepatic gene expression modulation (downregulation of lipogenic genes and upregulation of fat oxidative genes). Together, the findings of this work suggest that Bacillus toyonensis (strain SAU-20) can be a potential therapeutic strategy to modulate the microbiota and improve insulin sensitivity and hepatic steatosis in type 2 diabetic patients.

The pandemic of COVID-19 became the most important concern for patients with chronic diseases, especially diabetes mellitus (DM). The study of Zeng et al. aimed to explore the therapeutic action of 1,25-dihydroxy vitamin D [(1,25(OH)_2_D)] against COVID-19/DM. By combining network pharmacological analysis with molecular docking technology, authors identified hub targets, including EGFR, PIK3R1, PIK3CA, STAT3, and MAPK1, as well as the biological signaling pathways: HIF-1, FoxO, T cell receptor, PI3K, and Akt. The study showed that it is plausible to consider the 1,25(OH)_2_D as a strong binding affinity with these targets by forming hydrogen bonds and hydrophobic interactions, indicating the drug-protein interaction and the potential anti-COVID-19/DM activity of 1,25(301 OH)_2_D.

Severe COVID-19 is characterized by profound CD8^+^ T-cell dysfunction, which cannot be specifically treated to date. The study by Hirschberger et al. investigated whether metabolic CD8^+^ T-cell reprogramming by ketone bodies could be a promising strategy to overcome the immunoparalysis observed in COVID-19 patients. Flow cytometry and ELISA revealed elevated cytokine expression and secretion (up to + 24%) upon ketone treatment, as well as enhanced cell lysis capacity (+21%). Metabolic analyses using *Seahorse* technology revealed upregulated mitochondrial respiratory chain activity (+25%), enabling both elevated energy supply (+44%) and mitochondrial reactive oxygen species signaling. These beneficial effects of ketones might represent evolutionarily conserved mechanisms to strengthen human immunity.

In the work of Borges et al., the authors discuss the potential therapeutic effects of three compounds (melatonin, zinc, and vitamin C) as co-adjuvant treatments for COVID-19 patients, since previous studies have demonstrated some modulating effects on the immune system, oxidative stress, and/or virus infection by these compounds. The authors found some evidence that melatonin, zinc, and vitamin C can have some beneficial effects on COVID-19 patients in reducing hyperinflammation and oxidative stress. However, due to the limited studies and absence of large-scale clinical trials about the use of these compounds on COVID-19 patients, it is needed further studies to completely understand the potential therapeutic effects of these molecules on the treatment of COVID-19.

Zhao et al. used Mendelian randomization to evaluate the causal relationship among several factors, including sleep traits, body fat accumulation, glycemic traits, and gastroesophageal reflux disease (GERD). The authors analyzed genetic variants from more than 400,000 patients from published genome-wide association studies (GWASs). Interestingly, the authors found a causal relationship between sleep duration and insomnia and GERD risk, but no association between body fat accumulation or glycemic traits and GERD risk. Thus, findings suggest that improving sleep quality can be an important strategy to decrease the risk of the development GERD.

Studies about dietary nutrients and interventions on insulin resistance and inflammation have been widely advanced in the last decades. The studies published in the present Research Topic have further advanced in knowledge and the comprehension of the mechanisms involved in this process.

## Author contributions

SH, RG, RC, CL, and GNM-N prepared the first draft, critically reviewed it, and edited the manuscript. All authors have read, reviewed, and approved the final manuscript.
